# Muscle morphology and function following a transfemoral amputation: A systematic review

**DOI:** 10.33137/cpoj.v9i1.47289

**Published:** 2026-05-20

**Authors:** C.M. Kroes, R. Schelhaas, R. Dekker, J.H.B. Geertzen, K. van Kammen, H. Houdijk

**Affiliations:** 1 Department of Human Movement Sciences, University Medical Center Groningen, University of Groningen, Groningen, The Netherlands.; 2 Department of Anesthesiology, University Medical Center Groningen, University of Groningen, Groningen, The Netherlands.; 3 Department of Rehabilitation Medicine, University Medical Center Groningen, University of Groningen, Groningen, The Netherlands.; 4 Occupational Therapy, Hanze University of Applied Sciences, Groningen, The Netherlands.

**Keywords:** Transfemoral Amputation, Residual Limb, Muscle Atrophy, Muscle Volume, Muscle Torque, Muscle Power, Muscle Strength, Fatty Infiltration

## Abstract

**BACKGROUND:**

Restoring function and mobility after transfemoral amputation (TFA) is crucial for prosthesis use and heavily relies on muscle morphology and function in the residual limb. After amputation, the loss of distal muscle insertions alters morphology and might impair (muscle) functioning.

**OBJECTIVE:**

To provide a comprehensive and critically appraised overview of the existing evidence on muscle morphology and function of the residual limb following TFA.

**METHODOLOGY:**

A literature search was conducted in PubMed, Embase, and CINAHL, including articles published up to September 15, 2025. Language was restricted to English, Dutch and German. Articles were eligible if they included individuals with a TFA and objectively measured outcomes related to muscle morphology and/or function. Methodological quality was assessed using the Joanna Briggs Institute critical appraisal checklist and the review followed the PRISMA guidelines.

**RESULTS:**

Out of 1832 identified articles, 24 met the inclusion criteria. Eight articles reported outcomes related to muscle morphology while sixteen articles reported outcomes on muscle function. All articles assessing muscle volume and cross-sectional area reported reductions in most residual-limb muscle groups compared to the intact limb. Across articles assessing muscle function, the residual limb generated reduced hip flexion, extension, and abduction torques compared to the intact and/or control limb, while adduction torque diminished only at lower isokinetic speeds. The magnitude of alterations varied greatly between articles. No article investigated both muscle morphology and function. Therefore, the interrelationship between these outcomes could not be determined.

**CONCLUSION:**

The literature shows that muscle morphology and function are significantly altered in the residual limb following TFA, although reported effects vary across articles and muscle groups. Study heterogeneity limited exploration of factors that could account for this variation. This needs future study. Future research also should combine assessments of muscle morphology and function to better understand these variations.

## INTRODUCTION

Lower limb amputations (LLAs) represent a significant proportion of amputations worldwide, with worldwide annual incidence rates estimated between 5.8 and 31 per 100.000, of which about 26% are transfemoral amputations (TFAs).^1,2^

In the United States and Western Europe, over 90% of the major LLAs, including all amputations proximal to the ankle joint, are attributed to vascular disease.^3^ Other causes include trauma, tumors, infections, and congenital deficits.^4,5^ A major consequence of amputation is the loss of anatomical structures, including bones, skeletal muscles, and joints, which inevitably impairs an individual’s mobility.^6–8^

Rehabilitation following TFA often aims to restore impaired mobility, most frequently achieved through the provision of a prosthesis.^9,10^ The ability of an individual to walk with a prosthesis partly depends on the strength and control of the muscles in the residual limb. For example, greater hip muscle strength has been associated with improved balance and mobility in lower limb prosthetic users.^9,11^ Additionally, for individuals who do not use a prosthesis, rehabilitation and maintaining residual limb strength and control is important for performing daily activities and transfers.^12,13^ Therefore, adequate functioning of the residual limb muscles is important for daily functioning after TFA.

Following TFA, structural changes at the skeletal muscle level alter both the morphology and function of the muscles in the residual limb. Muscles that cross the level of amputation lose their insertions at the distal bone end. Following transection, the muscles may be re-attached or left unfixed.^14^ Re-attachment can be achieved in two ways: by anchoring the muscles directly to the bone with a myodesis, or by suturing them to the antagonistic muscle groups with a myoplasty.^14^ Both the amputation itself and the method of distal muscle fixation influence the mechanical properties of the muscles.^14–16^ The way muscles are re-attached influences their line of action and moment arm, which affects their contribution to movement and force production. Furthermore, studies argue that the absence of muscle fixation impairs muscle function and causes muscles to retract and atrophy.^17,18^ Additionally, a TFA may reduce the mechanical load on muscles and bones at the amputation site, which in turn can lead to muscle atrophy and decreased bone mineral density.^6,19–21^ Altogether, these structural muscle changes may cause muscle weakness and a subsequent decline in muscle function following TFA.^22,23^

Although it is important to understand structural muscle changes and their functional consequences following TFA, current literature lacks a comprehensive overview of both muscle morphology and muscle function and especially how these outcomes interrelate. Obtaining insights into how adaptations in muscle morphology and structure following TFA relate to muscle functioning is important not only for rehabilitation goals, but also for surgical decision making. While research on restoring mobility following TFA has largely focused on the capacity of prostheses, limited attention has been given to the capacity of the muscles in the residual limb. Therefore, this systematic review aims to provide a comprehensive and critically appraised overview of the existing evidence on both muscle morphology and function in the residual limb following TFA, and to better understand their interrelationships.

## METHODOLOGY

This systematic review was conducted and reported according to the Preferred Reporting Guidelines for Systematic Reviews and Meta-Analyses (PRISMA),^24^ and was registered in PROSPERO [CRD420251248737].

### Search Strategy

A systematic search was conducted by the first author (C.K) in the PubMed, Embase, and CINAHL databases, up to September 15, 2025. The search string was created using keywords related to the population and specific outcomes like muscle morphology and muscle function. The full search string is provided in **Appendix**.

### Study Selection

Articles were eligible for this systematic review if they:

- included individuals with TFA,
- objectively measured outcomes related to muscle morphology and/or muscle function,
- reported outcomes for both the residual limb and the intact limb or a control limb,
- were published in English, Dutch or German language,
- were full-text journal articles.

Articles were excluded if they:

- contained a systematic review or meta-analysis,
- included only individuals after osseointegration.

No restrictions were set on the publication date and no exclusion criteria based on the etiology of amputation or study design were applied. Muscle morphology outcomes included muscle volume, muscle or fat cross-sectional area, muscle atrophy, and fatty infiltration. Muscle function outcomes included muscle strength, muscle power, and muscle torque. Articles were required to report the outcomes of the intact limb or a control limb to put the muscle characteristics of the residual limb into perspective. Two authors (C.K and R.S) independently applied the in-and exclusion criteria during title, abstract and full-text screening. After the title and abstract screening, as well as after the full-text screening, any disagreements were resolved in a discussion meeting accompanied by a third researcher (K.K). Interrater reliability during the title/abstract screening and full-text screening was evaluated by calculating the percentage of agreement and Cohen’s kappa.^25,26^ Reference lists of included articles and relevant reviews were screened by one author (C.K) to ensure no articles were missed by the initial search.

### Data Extraction

Two authors (C.K and R.S) independently performed the data extraction and quality assessment. The selected outcomes were extracted and summarized into three tables: one with the study characteristics (study design, sample size, sex, age, etiology, and time since amputation); one with study outcomes related to muscle morphology (muscle volume, muscle and fat cross-sectional area, muscle atrophy, and fatty infiltration); and one with study outcomes related to muscle function (muscle torque, muscle power, and total work). For articles that included both individuals with a TFA and transtibial amputation (TTA), only the findings related to TFA were extracted. When separate reporting was not possible, total group results were presented. If outcomes were available only in figures, the authors were contacted to request the underlying data. One contact attempt was made in December 2025. In instances where data were not provided before May 2026, outcomes were marked as not reported, and the direction of the difference between the residual limb and the intact or control limb was qualitatively noted. Direction was defined as a residual-limb value differing from the intact or control limb by more than ±10%. The direction of the difference was also documented for articles that did not statistically test between-limb differences or did not report residual-limb values as a percentage of the intact or control limb. The ±10% threshold was used as a pragmatic, descriptive criterion to aid interpretation of small numerical differences across studies. This threshold was not intended to represent a statistically or clinically meaningful cutoff but was applied solely to provide consistent directional interpretation where only minimal increases or decreases were observed.

### Quality Assessment

Because no exclusion criteria were applied based on study design, the Joanna Briggs Institute (JBI) critical appraisal checklist for analytical cross-sectional articles was used to assess methodological quality.^27^ Two items from this checklist ("3. Were objective, standard criteria used for measurement of the condition?” and “4. Was the exposure measured in a valid and reliable way?”) were considered not applicable, as the included articles did not involve the assessment of a specific condition or exposure. Therefore, these two items were excluded from the assessment. Items concerning the reliability and validity of measurements were evaluated in light of contemporary methodological expectations. Several included studies used methods or reporting approaches that are now considered insufficient. Although acceptable at the time, these do not meet present-day standards and were accordingly rated as poor methodological quality for these items (scored as “–”).

## RESULTS

**Figure 1:**
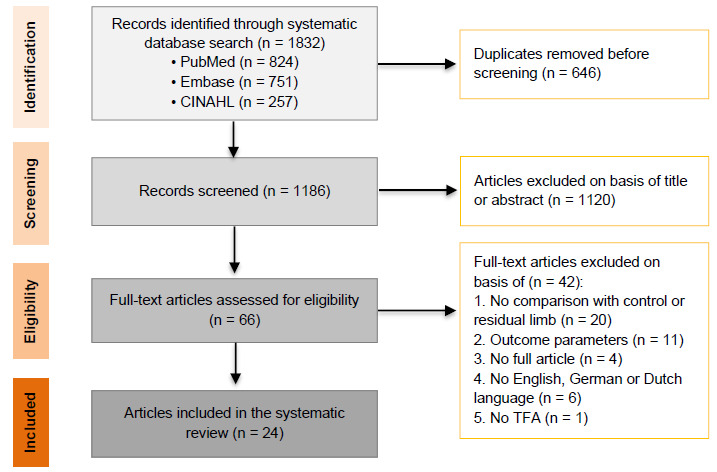
PRISMA flow diagram.

A total of 1832 articles were retrieved. After removing duplicates, 1186 titles and abstracts were screened, resulting in the exclusion of 1120 articles. Full texts of the remaining 66 articles were assessed for eligibility. Of these, 42 articles were excluded based on the inclusion and exclusion criteria, resulting in 24 included articles for this systematic review. **Figure 1** presents an overview of this screening procedure. Screening the reference lists of included articles and relevant reviews did not yield any additional studies. Reviewer agreement for title and abstract screening was 96.2%, with a Cohen’s Kappa of 0.53 (moderate agreement). Reviewer agreement for full text screening was 98.5%, with a Cohen’s Kappa of 0.968 (almost perfect agreement).

### Study Characteristics

The study characteristics of the included articles are presented in **Table 1**. The articles were published between 1973 and 2025. With regard to the amputation groups presented, 13 articles included only individuals with a TFA or allowed extraction of findings specifically related to the TFA group.^17,28–39^ Six articles included both individuals with a TFA and TTA,^40–45^ and three articles included individuals with various types of LLAs.^46–48^ Two articles included individuals with a bilateral amputation: one article involved bilateral TFAs and bilateral knee disarticulations,^49^ while the other included a mix of different bilateral amputation levels ranging from Symes to TFA.^50^ Relevant study sample sizes, defined as the number of participants with outcomes within the scope of this review, ranged from 5 to 56, yielding 482 participants in total, of whom 381 with an LLA. Thirteen articles included both males and females, eight articles included only males, and in three articles sex was not reported. The etiology between articles varied. Nine articles included only non-vascular patients, including trauma, tumor, infection, bone abscess, and congenital deficit causes. Seven articles included both vascular and non-vascular patients, and eight articles did not report etiology. Most of the articles (n = 21) recorded time since amputation, which ranged between 1 month and 48 years. Cross-sectional designs comprised the majority of the included studies, with only two articles incorporating pre-post comparisons.^36,43^

**Table 1:** Study Characteristics.

Author	Study Design	Relevant Study Sample Size (n)^1^	Type of Amputation	Sex (M)	Age (Years) Mean ± SD	Etiology of Amputation	Time Since Amputation
**Articles Reporting Muscle Morphology**							
Erikson et al.^34^	CS^2^	TFA (16)		16 M	43 ± 12	NR	15 ± 10 yrs
Finco et al.^46^	CS^2^	Amp (10) Control (20)	TFA (3), BTFA (1) TTA (5), BTTA (1) Healthy (10) Diabetic (10)	10 M 10 M 10 M	62 ± 11 58 ± 11 54 ± 9	NR	NR
Henson et al.^49^	CS^2^	Amp (6) Control (6)	BTFA^3^ NA	6 M 6 M	32 ± 3 34 ± 5	NR	7 ± 1 yrs^4^ (2-34 yrs)
Jaegers et al.^17^	CS^2^	Amp (12)	TFA	12 M	38 ± 12	Trauma (7) Tumor (5)	9 ± 9 yrs^4^ (2-35 yrs)
Levy et al.^36^	Pre- post	Amp (42)	TFA	30 M	60 ± 17^5^	Tumor (6), Infection (20), Vascular (16)	4 ± 3 yrs
Roda et al.^30^	CS	Amp (8)	TFA	2 M	47 ± 15	Non-vascular (8)	21 ± 15 yrs
Sherk et al.^39^	CS RM	Amp (5) Control (5)	TFA NA	NR	39 ± 11 39 ± 11	NR	20 ± 11 yrs
Yun et al.^33^	CS	Amp (5)	TFA	5 M	49 ± 11^6^	NR	7 ± 5 yrs^7^
**Articles Reporting Muscle Function**							
Butowicz et al.^37^	CS^2^	Amp (10) Control (8)	TFA NA	10 M 8 M	38 ± 6 29 ± 9	Trauma	11 ± 9 yrs^8^
Croisier et al.^40^	CS^2^	Amp (33)	TFA (14), TTA (19)	25 M	M: 59 ± 12 F: 64 ± 20	Vascular (23), Diabetes (10), Trauma (3), Tumor (2)	1 month - 10 yrs
Crozara et al.^48^	CS	Amp (17)	TFA (6), KD (4), TTA (7)	16 M	56 ± 15	Vascular (9), Trauma (8)	5 ± 3 yrs^8^
Demir et al.^41^	CS	Amp (32)	TFA (10), TTA (22)	32 M	34 ± 9	Trauma (32)	6 ± 9 yrs
Heitzmann et al. [A]^35^	CS	TFA (1) Control (17)	TFA NA	1 M 8 M	56 M: 53 ± 12 F: 56 ± 9	Trauma (1)	26 yrs
Heitzmann et al. [B]^28^	CS^2^	Amp (13) Control (18)	TFA NA	13 M 9 M	38±13 44±13	Tumor (2), Trauma (9), Bone abscess (1), Congenital (1)	15 ± 13 yrs^4^
James et al.^29^	CS^2^	Amp (38) Control (25)^9^	TFA NA	38 M 25 M	43 ± 13 24 (20-28)	NR	18 yrs (2-48 yrs)
Leijendekkers et al.^47^	CS	Amp (44)^10^	TFA (35), KD (1), TTA (7), Foot (1)	28 M	54 ± 13	NR	NR
Lin et al.^42^	CS	Amp Fall (6) Amp nFall (7) Control (13)	TFA (2), TTA (4) TFA (2), TTA (5) NA	3 M 6 M 2 M	65 ± 16 53 ± 18 29 ± 7	NR	5 ± 3 yrs 28 ± 18 yrs
Nolan et al.^43^	Pre- post	Amp TG (7) AMP CG (8)	TFA (4), TTA (3) TFA (5), TTA (3)	6 M 5 M	41 ± 8 49 ± 9	Trauma (5), Tumor (1) Congenital (1) Trauma (8)	8 ± 9 yrs 8 ± 11 yrs
Rutkowska-Kucharska et al.^31^	CS^2^	Amp (8)	TFA	NR	44 ± 14^6^	Trauma (6), Congenital (2)	NR
Ryser et al.^32^	CS^2^	Amp (10) Control (10)	TFA NA	8 M 8 M	41 ± 13 42 ± 15	Trauma (7), Tumor (2), Vascular (1)	8 yrs (4 months - 17 yrs)
Sawers & Fatone [A]^44^	CS	Amp (26)	TFA (13), TTA (13)	13 M	54	Non-vascular (19) Vascular (7)	Median: 12 yrs MAD: 13 yrs
Sawers & Fatone [B]^45^	CS	Amp (28)^11^ Control (28)	TFA (14), TTA (14) NA	NR NR	Median: 55 Median: 55	Non-vascular (21) Vascular (7)	Median: 12 yrs IQR: 17 yrs
Sawers & Fatone [C]^38^	CS	Amp (13)^11^	TFA	6M	52 ± 17^6^	Non-vascular (9) Vascular (4)	14 ± 12 yrs^4^ (3 - 38 yrs)
Visser et al.^50^	CS	Amp BLLA (10) Control (11)	TFA/TTA (4), TTA/TTA (3), Sym/Sym (3) NA	8 M 7 M	44 ± 11 44 ± 12	Trauma (2), Infection (4), Congenital (4)	20 ± 19 yrs

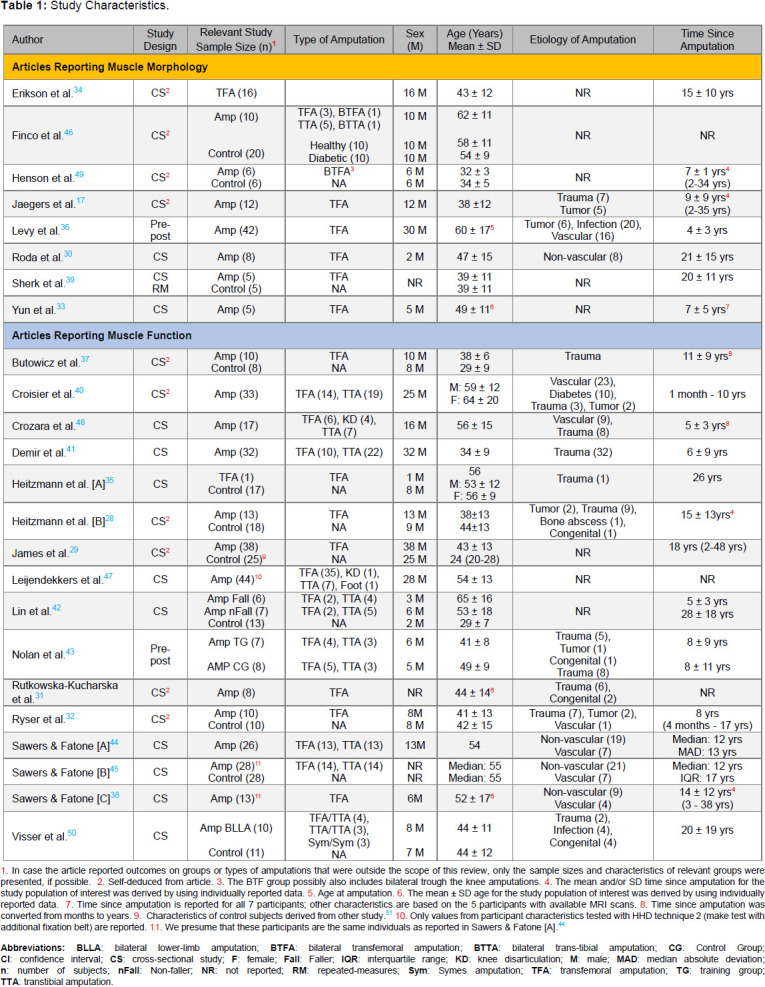

1. In case the article reported outcomes on groups or types of amputations that were outside the scope of this review, only the sample sizes and characteristics of relevant groups were presented, if possible.

2. Self-deduced from article.

3. The BTF group possibly also includes bilateral trough the knee amputations.

4. The mean and/or SD time since amputation for the study population of interest was derived by using individually reported data.

5. Age at amputation.

6. The mean ± SD age for the study population of interest was derived by using individually reported data.

7. Time since amputation is reported for all 7 participants; other characteristics are based on the 5 participants with available MRI scans.

8. Time since amputation was converted from months to years.

9. Characteristics of control subjects derived from other study.^51^

10. Only values from participant characteristics tested with HHD technique 2 (make test with additional fixation belt) are reported.

11. We presume that these participants are the same individuals as reported in Sawers & Fatone [A].^44^

**Abbreviations: BLLA:** bilateral lower-limb amputation; **BTFA:** bilateral transfemoral amputation; **BTTA:** bilateral transtibial amputation; **CG:** Control Group; **CI:** confidence interval; **CS:** cross-sectional study; **F:** female; **Fall:** Faller; **IQR:** interquartile range; **KD:** knee disarticulation; **M:** male; **MAD:** median absolute deviation; **n:** number of subjects; **nFall:** Non-faller; **NR:** not reported; **RM:** repeated-measures; **Sym:** Symes amputation; **TFA:** transfemoral amputation; **TG:** training group; **TTA:** transtibial amputation.

### Quality Assessment

**Table 2** presents the quality assessment using the JBI critical appraisal checklist. Three articles met all six criteria and were therefore rated as high quality.^36,41,48^ Twelve articles met five of the six criteria,^17,31,33,35,38–40,44–47,50^ mostly due to incomplete descriptions of the study population and/or study setting. In most cases, only the time period or location of study setting was missing, whereas three articles lacked additional information on sex, cause of amputation and/or time since amputation.^31,46,47^ Four articles fulfilled four criteria,^28,30,32,42^ indicating moderate methodological quality, whereas three articles met with only three^37,43,49^ and two articles met with only two criteria,^29,34^ indicating low methodological quality. Articles that met four or fewer criteria commonly showed multiple limitations, including inadequate reporting of study subjects and settings, insufficient consideration of confounders, and/or concerns about the validity and reliability of outcome measurements. Measurement validity and reliability were judged questionable in five articles, primarily because older measurement systems or methods were used,^29,32,34,43^ and in one article due to unreliable methos for positioning of the thigh.^37^ These assessments reflect our decision to evaluate the measurements according to current research standards and methodology. The majority of articles used appropriate statistical analysis, although they did not consistently correct for multiple comparisons.

**Table 2:** JBI critical appraisal checklist for analytical cross-sectional studies.

Reference	1. Were the criteria for inclusion in the sample clearly defined?	2. Were the study subjects and the setting described in detail?	3. Was the exposure measured in a valid and reliable way?	4. Were objective, standard criteria used for measurement of the condition?	5. Were con-founding factors identified?	6. Were strategies to deal with confounding factors stated?	7. Were the outcomes measured in a valid and reliable way?	8. Was appropriate statistical analysis used?	QA Score
Butowicz et al.^37^	+	−^1^			+	−	−	+	**3**
Croisier et al.^40^	+	−^1^			+	+	+	+	**4**
Crozara et al.^48^	+	+			+	+	+	+	**6**
Demir et al.^41^	+	+			+	+	+	+	**6**
Erikson et al.^34^	−	**-**			+	−	−	+	**2**
Finco et al.^46^	+	**-**			+	+	+	+	**5**
Heitzmann et al. [A]^35^	+	−^1^			+	+	+	+	**5**
Heitzmann et al. [B]^28^	+	−^1^			+	−	+	+	**4**
Henson et al.^49^	−	−			+	+	+	−	**3**
Jaegers et al.^17^	+	−^1^			+	+	+	+	**5**
James et al.^29^	−	−			+	−	−	+	**2**
Leijendekkers et al.^47^	+	−			+	+	+	+	**5**
Levy et al.^36 2^	+	+			+	+	+	+	**6**
Lin et al.^42^	+	−^1^			+	−	+	+	**4**
Nolan et al.^43 2^	−	−			+	+	x	+	**3**
Roda et al.^30^	+	−^1^			+	−	+	+	**4**
Rutkowska-Kucharska et al.^31^	+	−			+	+	+	+	**5**
Ryser et al.^32^	+	−^1^			+	+	−	+	**4**
Sawers & Fatone [A]^44^	+	−^1^			+	+	+	+	**5**
Sawers & Fatone [B]^45^	+	−^1^			+	+	+	+	**5**
Sawers & Fatone [C]^38^	+	−^1^			+	+	+	+	**5**
Sherk et al.^39^	+	−^1^			+	+	+	+	**5**
Visser et al.^50^	+	−^1^			+	+	+	+	**5**
Yun et al.^33^	+	+			+	−	+	+	**5**

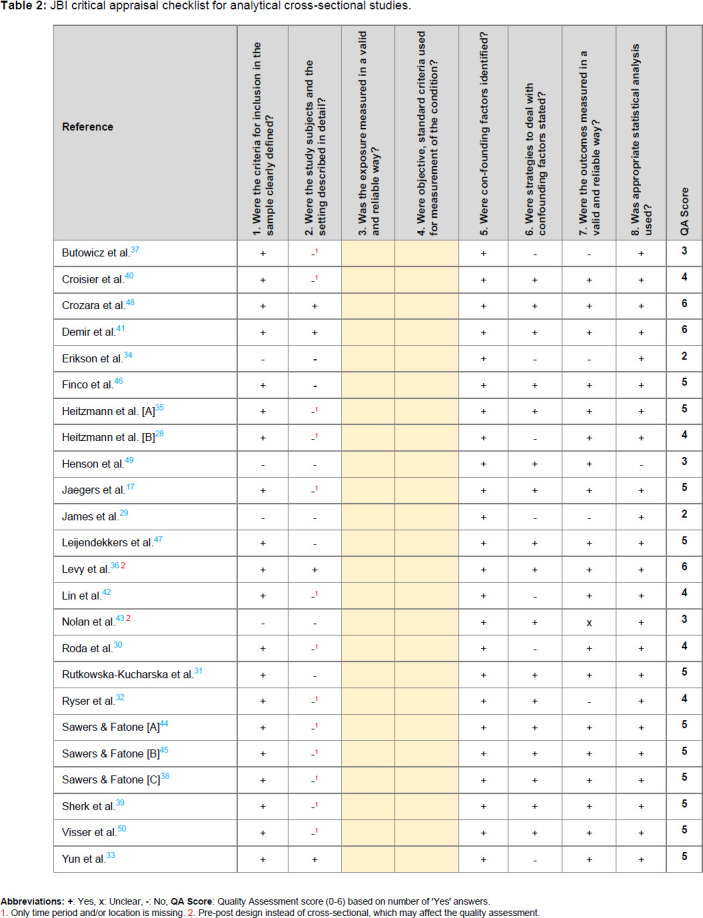

**Abbreviations: +:** Yes, **x:** Unclear, **-:** No, **QA Score:** Quality Assessment score (0-6) based on number of 'Yes' answers.

1. Only time period and/or location is missing.

2. Pre-post design instead of cross-sectional, which may affect the quality assessment.

### Study Outcomes on Muscle Morphology and Function

The outcomes of the articles included are presented in **Table 3** for muscle morphology and **Table 4** for muscle function. Eight articles reported outcomes related to muscle morphology^17,30,33,34,36,39,46,49^ and 16 articles reported outcomes related to muscle function.^28,29,31,32,35,37,38,40–45,47,48,50^ None of the included articles assessed both muscle morphology and muscle function.

**Table 3:** Summary of articles investigating muscle morphology in individuals with a TFA.

Author	Comparison	Measurement	Muscle Group	Outcome (Mean ± SD)		Residual vs. Intact (%)^1^	Control	Residual vs. Control (%)^1^
				Residual	Intact			
Erikson et al.^34^	Residual^2^ Intact	**MSCA** (% of total CSA of the thigh) **Tool:** X-ray	**Thigh** 5-10 cm proximal to stump end	54 ± 9%	75 ± 6%	45%***		
Finco et al.^46^	Residual^3^ Intact Control^4^	**MCSA** (mm^2^) **FCSA** (mm^2^) **Tool:** CT scan	**Thigh** At 50% of femur length **Thigh**^4^ At 50% of femur length	7792 ± 3334 7214 ± 5060	9398 ± 3620 6755 ± 4759	R < I^*^ R ≈ I ^ns^	12357 ± 2497^HC^ 10174 ± 2717^DC^ 8982 ± 7248^HC^ 9589 ± 6595^DC^	R < C^*^ R < C^*^ R ≈ C^ns^ R ≈ C^ns^
Sherk et al.^39^	Residual^2^ Intact Control	**MCSA** (mm^2^) **FSCA** (mm^2^) **Tool:** pQCT scan	**Thigh** At 5% of the RLL, proximal to stump end **Thigh**^4^ At 5% of the RLL, proximal to stump end	4819 ± 2812 8447 ± 2950	17123 ± 5448 9109 ± 4069	R < I^**^ R ≈ I ^ns^	17326 ± 5060 7361 ± 5801	R < C^**^ R > C^ns^
Henson et al.^49^	Residual^5^ Control	**Muscle Volume** (cm^3^) **Tool:** MRI scan	**ADD total** Add brevis Add longus Add magnus Gracilis Pectineus Sartorius **ABD total** GMED GMIN TFL ------------- GMAX ------------- Iliopsoas ------------- Med hamstr Lat hamstr ------------- RF ------------- Vastii ------------- Total	**ADD** **1190 ± 178** 165 ± 38 216 ± 25 621 ± 95 98 ± 31 91 ± 22 194 ± 38 **ABD** **712 ± 101** 470 ± 54 134 ± 32 108 ± 21 -------------------- 1266 ± 237 -------------------- 507 ± 58 -------------------- 308 ± 85 282 ± 114 -------------------- 240 ± 55 -------------------- 857 ± 184 -------------------- 555 ± 876			**1496 ± 177** 130 ± 149 249.5 ± 7813.8 791 ± 0 165 ± 82 79 ± 0 247 ± 0 **665 ± 145** 424 ± 824 130 ± 0 112 ± 0 ------------------------ 1253 ± 334 ------------------------ 457 ± 433 ------------------------ 624 ± 1876 453 ± 235 ------------------------ 428 ± 1287 ------------------------ 2144 ± 560 ------------------------ 8793 ± 1068	85 ± 13%^*^ 122 ± 28%^*^ 84 ± 10%^*^ 78 ± 12%^**^ 58 ± 18%^**^ 117 ± 28%^ns^ 77 ± 15%^**^ **106 ± 15%^ns^** 110 ± 13%^*^ 103 ± 25%^ns^ 95 ± 19%^ns^ --------------- 100 ± 19%^ns^ --------------- 110 ± 13%^**^ --------------- 49 ± 14%^**^ 62 ± 25%^**^ --------------- 56 ± 13%^**^ --------------- 39 ± 9%^**^ --------------- 65 ± 13%^**^
Jaegers et al.^17^	Residual^2^ Intact	**Muscle Volume** (cm^3^) **Tool:** MRI scan	Iliopsoas RF Sartorius GMAX Biceps fem SemiT SemiM GMED GMIN TFL ADD brevis ADD min ADD longus ADD magnus Gracilis Pectineus	**FLEX** 387 ± 63 124 ± 64 93 ± 46 **EXT** 801 ± 280 113 ± 79 59 ± 45 76 ± 62 **ABD** 331 ± 125 133 ± 110 66 ± 25 **ADD** 106 ± 38 82 ± 28 149 ± 64 366 ± 164 53 ± 21 60 ± 18	472 ± 59 289 ± 118 169 ± 65 1252 ± 284 399 ± 146 205 ± 50 283 ± 58 423 ± 93 184 ± 108 103 ± 27 118 ± 29 81 ± 29 168 ± 49 792 ± 157 120 ± 26 64 ± 15	R < I^NR^ R < I^NR^ R < I^NR^ R < I^NR^ R < I^NR^ R < I^NR^ R < I^NR^ R < I^NR^ R < I^NR^ R < I^NR^ R ≈ I^NR^ R ≈ I^NR^ R < I^NR^ R < I^NR^ R < I^NR^ R ≈ I^NR^	638 ± 55 309 ± 30 181 ± 16 968 ± 30 326 ± 42 197 ± 26 291 ± 26 299 ± 61 120 ± 16 87 ± 16 105 ± 5 68 ± 23 189 ± 10 515 ± 91 113 ± 16 73 ± 12	R < C^na^ R < C^na^ R < C^na^ R < C^na^ R < C^na^ R < C^na^ R < C^na^ R > C^na^ R > C^na^ R < C^na^ R ≈ C^na^ R > C^na^ R < C^na^ R < C^na^ R < C^na^ R < C^na^
Roda et al.^30^	Residual^2^ Intact	**Muscle volume** (mm^2^/kg) normalized by body mass and height **Fat infiltration** (%) **Tool:** MRI scan	**GMED** **GMED**	NR NR	NR NR	63.7%^NR,^^6^ 135.5%^NR,^^6^		
Yun et al.^33^	Residual^2,7^ Intact	**Muscle volume** (cm^3^) **Tool:** MRI-scan		**FLEX**^8^ 210 (159–214) **ADD**^8^ 730 (572–893) **EXT**^8^ 320 (298–412) **Knee EXT (vastii)**^8^ 514 (511–517) **Total**^8^ 1774 (1659–1887)	362 (314–363) 993 (939-1062) 608 (512–705) 1364 (1131–1393) 3358 (2856–3660)	R < I^**^ R ≈ I ^ns^ R < I^**^ R < I^**^ R < I^**^		
Levy et al.^36^	Residual^2^ Intact	**MCSA** (mm^2^) at the level of L3-4 disc space **Hounsfield units**^10^ **Tool:** CT scan	**Psoas** **Psoas**	881 ± 401 37 ± 17	996 ± 377 42 ± 17	R < I^**^ R < I^**^	1074 ± 453^9^ 43 ± 18^9^	R < C*** R < C^**^

* p<.05

** p<.01

*** p<.001

1. Percentages represent the residual limb as a proportion of the intact or control limb. If these percentages were not provided, the comparison is expressed qualitatively by indicating which limb exhibited the greater muscle or fat volume. If differences were not statistically tested, direction was defined as a residual-limb value differing from the intact or control limb by more than ±10%.

2. Unilateral transfemoral amputation.

3. Combination of unilateral and bilateral transfemoral and transtibial amputations.

4. Comparison to healthy and diabetic controls (HC and DC).

5. Bilateral transfemoral amputation.

6. The article reported percentage difference between the amputated and intact limb. Outcomes were expressed relative to the intact limb, with decreases in muscle volume represented as 100% − ∆muscle volume% and increases in fatty infiltration as 100% + ∆fatty infiltration%.

7. Two out of the seven participants were excluded from analysis.

8. Median and interquartile range, the article excluded the iliopsoas and gluteus maximus from the volumetric analysis.

9. Control limb values represent the pre‑amputation MCSA and pre-amputation Hounsfield units of the amputated leg.

10. These units serve as a proxy for fatty changes; decreased attenuation indicates increased atrophy.

**Abbreviations: ABD:** abductors; **ADD:** adductors; **CSA:** cross-sectional area; **CT:** computed tomography; **EXT:** extensors; **FCSA:** fat cross sectional area; **fem:** femoris; **FLEX:** flexors; **GMAX:** gluteus maximus; **GMED:** gluteus medius; **GMIN:** gluteus minimus; **hamstr:** hamstrings; **Lat:** lateralis; **MCSA:** muscle cross sectional area; **Med:** medialis; **min:** minimus; **MRI:** magnetic resonance imaging; **na:** not analyzed; **NR:** not reported; **ns:** not significant; **RF:** rectus femoris; **RLL:** residual limb length; **SemiM:** semimembranosus; **SemiT:** semitendinosus; **TFL:** tensor fascia latae.

**Table 4:** Summary of articles investigating muscle function in individuals with a TFA.

Author	Comparison	Measurement	Muscle Action and Hip Joint Position	Outcome (Mean ± SD)		Residual vs. Intact (%)^1^	Control	Residual vs. Control (%)^1^
				Residual	Intact			
Butowicz et al.^37^	Residual^2^ Intact Control	**Peak torque** (Nm) **Tool:** HHD – Lafayette	**Isometric** 10° abduction side lying	**ABD** NR	NR	R < I^4^	NR	R ≈ C^ns^
Croisier et al.^40^	Residual^3^ Intact	**Peak torque normalized to body mass** (Nm/kg) **Tool:** Isokinetic Dynamometer – KINTREX 1000	**Isokinetic** 30°/s 60°/s 120°/s 30°/s 60°/s 120°/s 30°/s 60°/s 120°/s 30°/s 60°/s 120°/s	**FLEX** 0.80 ± 0.34 NR NR **EXT** 0.66 ± 0.31 NR NR **ABD**^6^ 0.78 ± 0.34 NR NR **ADD**^6^ 0.76 ± 0.23 NR NR	1.04 ± 0.34* NR NR 1.00 ± 0.40* NR NR 0.98 ± 0.29* NR NR 0.87 ± 0.23* NR NR	78%^5^ R < I* R < I* 67%^5^ R < I* R < I* 77%^5^ R < I* R < I* 89%^5^ R ≈ I^ns^ R ≈ I^ns^		
Crozara et al.^48^	Residual^3^ Intact	**Peak torque and power normalized to body mass** (Nm/kg) & (Watts/kg) **Tool:** Isokinetic Dynamometer – Biodex System 4	**Isometric** 60° flexion 60° extension 15° abduction 15° adduction **Isokinetic** 60°/s 180°/s 60°/s 180°/s 30°/s 90°/s 30°/s 90°/s **Isokinetic Power** 60°/s 180°/s 60°/s 180°/s 30°/s 90°/s 30°/s 90°/s	**FLEX**^7^ 0.70 ± 0.31 **EXT**^7^ 1.81 ± 0.52 **ABD**^7^ 0.96 ± 0.40 **ADD**^7^ 1.25 ± 0.35 **FLEX**^7^ 0.85 ± 0.27 0.74 ± 0.25 **EXT**^7^ 1.15 ± 0.51 0.91 ± 0.42 **ABD**^7^ 0.94 ± 0.34 0.76 ± 0.32 **ADD**^7^ 1.14 ± 0.39 0.89 ± 0.40 **FLEX**^7^ 0.52 ± 0.19 0.78 ± 0.35 **EXT**^7^ 0.67 ± 0.31 0.90 ± 0.60 **ABD**^7^ 0.31 ± 1.12 0.57 ± 0.25 **ADD**^7^ 0.31 ± 0.14 0.43 ± 0.30	0.94 ± 0.25 2.23 ± 0.50 0.97 ± 0.33 1.27 ± 0.22 1.21 ± 0.26 1.09 ± 0.31 1.46 ± 0.37 1.13 ± 0.35 1.09 ± 0.30 0.89 ± 0.24 1.17 ± 0.24 1.01 ± 0.27 0.77 ± 0.18 1.26 ± 0.44 0.84 ± 0.26 1.05 ± 0.51 0.36 ± 0.11 0.66 ± 0.23 0.31 ± 0.10 0.55 ± 0.37	75%***^5^ 81%***^5^ R ≈ I^ns^ R ≈ I^ns^ 70%***^5^ 68%***^5^ 79%**^5^ 80%*^5^ 86%*^5^ 86%*^5^ R ≈ I^ns^ R ≈ I^ns^ 67%***^5^ 62%***^5^ 79%**^5^ R ≈ I^ns^ 80%*^5^ R ≈ I^ns^ R ≈ I^ns^ R ≈ I^ns^		
Demir et al.^41^	Residual^2^ Intact	**Peak torque and total work**^8^ **Tool:** Isokinetic Dynamometer – Cybex	**Isokinetic** 60°/s **Total work** 60°/s	**FLEX** 56.1 ± 24.5 **EXT** 61.0 ± 31.7 **FLEX** 63.5 ± 21.1 **EXT** 67.1 ± 34.0	67.3 ± 25.7 69.3 ± 17.5 80.8 ± 36.8 88.9 ± 56.0	R ≈ I^ns^ R ≈ I^ns^ R ≈ I^ns^ R ≈ I^ns^		
Heitzmann et al. [A]^35^	Residual^2^ Control	**Peak torque normalized to body mass** (Nm/kg) **Tool:** OpTIMo device	**Isometric** Vertical position of the thigh while standing	**FLEX** 0.66 **EXT** 0.72 **ABD** 0.64 **ADD** 0.95			1.19 ± 0.51 1.05 ± 0.34 1.17 ± 0.38 1.19 ± 0.38	R < C^na^ R < C^na^ R < C^na^ R < C^na^
Heitzmann et al. [B]^28^	Residual^2^ Control	**Peak torque normalized to body mass** (Nm/kg) **Tool:** OpTIMo device	**Isometric** Vertical position of the thigh while standing	**FLEX**^9^ 0.93 ± 0.40 **EXT**^9^ 1.11 ± 0.33 **ABD**^9^ 0.85 ± 0.25 **ADD**^9^ 0.87 ± 0.36			1.63 ± 0.73 * 1.30 ± 0.39^ns^ 1.41 ± 0.41*** 1.37 ± 0.42 **	57% 85% 60% 63%
James et al.^29^	Residual^2^ Intact	**Muscle torque** (kilopond metres, kpm) **Tool:** Dynamometer	**Isometric** Neutral position of the hip joint while standing	**FLEX**^10^ 10.7 ± 0.76 **EXT**^10^ 9.3 ± 0.56 **ABD**^10^ 8.2 ± 0.50 **ADD**^10^ 7.2 ± 0.46 **Total**^10^ 35.4 ± 2.09	20.5 ± 0.72^ns^ 16.7 ± 0.82 ** 14.9 ± 0.61^ns^ 13.7 ± 0.66 ** 65.8 ± 2.60^ns^	52% 57% 54% 53% 54%	20.2 ± 0.62 20.0 ± 0.74 15.2 ± 0.57 17.0 ± 0.80 72.4 ± NR	R < C^na^ R < C^na^ R < C^na^ R < C^na^ R < C^na^
Leijendekkers et al.^47^	Residual^3^ Intact	**Peak torque** (Nm) **Tool:** HHD – microFET2	**Isometric** Neutral – supine Test^11^ Retest^11^	**ABD** 56.1 ± 22.9 57.8 ± 23.3	67.0 ± 24.5^na^ 69.4 ± 24.6	89%^5^ R < I^na^		
Lin et al.^42^	Residual^3^ Control	**Muscle power** (W/s) **Tool:** Isokinetic Dynamometer – Biodex System 4	**Isokinetic** 60°/s 120°/s 60°/s 120°/s 60°/s 120°/s 60°/s 120°/s	**FLEX**^nFall^ 48.1 ± 15.3 58.2 ± 16.8 **EXT**^nFall^ 43.1 ± 14.7 53.8 ± 15.6 **FLEX**^Fall^ 32.5 ± 21.2 28.8 ± 18.5 **EXT**^Fall^ 32.0 ± 29.7 37.7 ± 40.5	47.2 ± 17.4 62.8 ± 28.6 50.4 ± 15.7 70.4 ± 28.2 25.3 ± 17.3 25.4 ± 21.3 27.3 ± 18.6 31.5 ± 27.0	R ≈ I^na^ R ≈ I^na^ R < I^na^ R < I^na^ R > I^na^ R > I^na^ R > I^na^ R > I^na^	54.1 ± 22.4 66.9 ± 33.9 58.2 ± 21.2 80.5 ± 41.3 54.1 ± 22.4 66.9 ± 33.9 58.2 ± 21.2 80.5 ± 41.3	R < C^na^ R < C^na^ R < C^na^ R < C^na^ R < C^na^ R < C^na^ R < C^na^ R < C^na^
Nolan et al.^43^	Residual^2^ Intact	**Peak torque normalized** (Nm/kg) **Tool:** Isokinetic Dynamometer – SPARK system	**Isokinetic** 60°/s 120°/s 60°/s 120°/s 60°/s 120°/s 60°/s 120°/s	**FLEX**^TG^ NR NR **EXT**^TG^ NR NR **FLEX**^CG^ NR NR **EXT**^CG^ NR NR	NR NR NR NR NR NR NR NR	R ≈ I^4^ R ≈ I^4^ R < I^4^ R < I^4^ R ≈ I^4^ R < I^4^ R < I^4^ R < I^4^		
Rutkowska-Kucharska et al.^31^	Residual^2^ Intact	**Peak torque normalized** (Nm/kg) **Tool:** Isokinetic Dynamometer – Biodex System 4	**Isometric** Position unknown^12^ **Isokinetic** 60°/s 120°/s 60°/s 120°/s	**FLEX** 1.00 ± 0.15 **EXT** 1.32 ± 1.44 **FLEX** 0.93 ± 0.15 0.77 ± 0.15 **EXT** 1.11 ± 0.97 0.90 ± 0.78	1.28 ± 0.21 1.77 ± 0.65 1.37 ± 0.21 1.14 ± 0.15 1.58 ± 0.24 1.14 ± 0.24	R < I* R < I* R < I* R < I* R < I* R < I*		
Ryser et al.^32^	Residual^2^ Intact Control	**Peak torque** (Nm) with and without gravity correction^13^ **Tool:** Isokinetic Dynamometer - Modified Cybex II	**Isometric** Neutral **Isokinetic** 30°/s 90°/s 150°/s	**ABD** 62 ± 32 **ABD** 42 ± 23 29 ± 17 24 ± 14	86 ± 41 ** 67 ± 32 60 ± 34 NR	70% R < I^na^ R < I^na^	85 ± 27^ns^ 71 ± 25^na^ 65 ± 25^na^ NR	71% 59% 45%
Sawers & Fatone [A]^44^	Residual^3^ Intact	**Peak torque** (Nm) **Tool:** Isokinetic Dynamometer – Biodex System 4	**Isometric** 20° flexion 10° abduction	**EXT**^14^ 72.9 ± 17.8 **ABD**^14^ 81.4 ± 12.0	71.4 ± 12.2 74.3 ± 17.6	R ≈ I^na^ R ≈ I^na^		
Sawers & Fatone [B]^45^	Residual^3^ Intact Control	**Peak torque normalized** to body mass × thigh length (Nm/(kg × TL)) and log-transformed **Tool:** Isokinetic Dynamometer – Biodex System 4	**Isometric** 20° flexion 20° extension 10° abduction 10° adduction	**FLEX**^14,15^ 1.23 ± 0.14 **EXT**^14,15^ 1.30 ± 0.10 **ABD**^14,15^ 1.40 ± 0.12 **ADD**^14,15^ 1.12 ± 0.11	0.40 ± 0.12 0.93 ± 0.11 0.35 ± 0.10 0.30 ± 0.11	R > I*** R > I*** R > I*** R > I***	1.27 ± 0.08 1.35 ± 0.07 1.32 ± 0.09 0.92 ± 0.07	R ≈ C^ns^ R ≈ C^ns^ R ≈ C^ns^ R > C***
Sawers & Fatone [C]^38^	Residual^2^ Intact	**Peak torque normalized** to body mass × thigh length (Nm/(kg × TL)) **Tool:** Isokinetic Dynamometer – Biodex System 4	**Isometric peak** 20° flexion 20° flexion 10° abduction 10° adduction	**FLEX**^16^ 17.3 (13.1–21.4) **EXT**^16^ 25.9 (22.1–29.6) **ABD**^16^ 25.3 (20.4–30.2) **ADD**^16^ 13.9 (11.7–16.2)	2.42 (1.97–2.86) 8.06 (6.81–9.31) 2.08 (1.75–2.41) 1.71 (1.43–1.98)	R > I^na^ R > I^na^ R > I^na^ R > I^na^		
Visser et al.^50^	Residual^17^ Control	**Peak torque normalized** to body weight (Nm/kg) **Tool:** Isokinetic Dynamometer – KinCom	**Isokinetic** **Eccentric** 30°/s Standing **Concentric** 30°/s Standing **Average peak torque**	**FLEX** NR **EXT** NR **ABD** NR **FLEX** NR **EXT** NR **ABD** NR **Total** NR			NR NR NR NR NR NR NR	R ≈ C^ns^ R ≈ C^ns^ R ≈ C^ns^ R ≈ C^ns^ R < C* R ≈ C^ns^ 66–71%^na^^5^

* p<.05

** p<.01

*** p<.001

1. Percentages represent the residual limb as a proportion of the intact or control limb. If percentages were not provided, the comparison is expressed qualitatively by indicating which limb exhibited the greater muscle torque, power or work. If differences were not statistically tested, direction was defined as a residual-limb value differing from the intact or control limb by more than ±10%.

2. Unilateral transfemoral amputation.

3. Combination of unilateral transfemoral and unilateral transtibial and/or trough knee amputation.

4. Direction derived from visual inspection of figures, no statistical analysis performed.

5. The article reported percentage deficit or difference. Values expressed relative to the intact/control limb were calculated as 100% − deficit%.

6. Abduction and adduction were assessed in only 8 of the 33 participants, with unclear distribution across TTA and TFA.

7. Mean and SD were derived from the raw dataset shared by the authors.

8. Measurement units not specified.

9. Adjusted significance level of p < .0125 following Bonferroni correction.

10. Mean ± standard error.

11. Only values from rater 1 using HHD technique 2 (make test with additional fixation belt) are reported.

12. Although the authors report that the testing position was derived from the Biodex System 4 Pro manufacturer’s manual, we were unable to identify this position.

13. Only values corrected for gravity are reported.

14. Median ± median absolute deviation.

15. Bonferroni adjusted alpha level of 0.0167.

16. Mean (95% confidence interval).

17. Bilateral transfemoral and transtibial amputation.

**Abbreviations: C:** control limb; **CG:** control group; **Fall:** faller; **HHD:** hand-held dynamometer; **I:** intact limb; **na:** not analyzed; **nFall:** non-faller; **NR:** not reported; **ns:** not significant; **OpTIMo:** Optimal Testing Isometric Moment; **R:** residual limb; **TG:** training group (pre-training values only).

### Muscle Morphology

Eight articles reported study outcomes related to muscle morphology (**Table 3**). X-rays, CT scans and MRI scans, were used to measure muscle or fat cross-sectional area, fatty infiltration and muscle volume or muscle atrophy.

### Muscle Cross-Sectional Area

All articles assessing thigh muscle cross-sectional area (MCSA) demonstrated a significant reduction in total MCSA in the residual limb compared to the intact limb^34,39,46^ or control limb.^39,46^ Additionally, Levy et al.^36^ reported a significant lower MSCA in the psoas muscle of the residual limb compared to the intact and control limb.

### Fat Cross-Sectional Area and Fatty Infiltration

Two articles assessing thigh fat cross-sectional area (FSCA) reported non-significant differences between the residual limb and the intact or control limb.^39,46^ However, for the gluteus medius and psoas muscles, the residual limb showed increased fatty infiltration^30^ and reduced Hounsfield units^36^ compared to the intact limb. A similar reduction in Hounsfield units was observed in the psoas muscle when comparing the residual limb with a control limb.^36^

### Muscle Volume

Three articles compared muscle volumes between the residual and intact limb.^17,30,33^ Jaegers et al.^17^ reported atrophy across all hip muscles of the residual limb compared with the intact limb, although the degree of atrophy varied between muscles. The smallest percentages of atrophy (11.5-23.6%) were observed in the pectineus, iliopsoas, gluteus minimus, and the adductor minimus, brevis, and longus. In contrast, the sartorius, gracilis, hamstrings, adductor magnus, and tensor fascia latae exhibited the greatest percentages of atrophy (40.3-72.8%).^17^ Yun et al.^33^ observed significantly lower muscle volumes in the flexors, extensors and vastii of the residual limb compared to the intact limb, while noting no significant difference for the adductors.^33^ Similarly, Roda et al.^30^ reported that the gluteus medius volume of the residual limb was 36.3% smaller than that of the intact limb, after normalizing to body mass and height. One article compared the muscle volume of the residual limb in bilateral transfemoral amputees with that of controls and found a 34.8% reduction in total muscle volume in the residual limb.^49^ This overall decrease was reflected in statistically significant reductions in the hip adductors (15.2%), medial hamstrings (50.8%), lateral hamstrings (38.5%), and vastus muscles (60.6%). However, the muscle volume of the hip abductors and the iliopsoas showed significant increases of 6.3% and 9.9%, respectively.^49^

### Muscle Function

Muscle function was evaluated using an isokinetic dynamometer, a static dynamometer in standing position, or a hand-held dynamometer (HHD), see **Table 4**. The outcomes of interest included peak hip torque, power, and total work, which will be discussed separately.

### Muscle Torque

Articles assessing hip muscle torque following TFA reported outcomes for the residual limb and the intact limb,^31,37,38,40–44,47,48^ the residual limb and a control limb,^28,35,50^ or both.^32,42,45^ Results are presented separately for hip flexion and extension torque and hip abduction and adduction torque.

### Hip Flexion and Extension

Six of the nine articles that reported hip flexion and extension torques for the residual and intact limb performed direct comparisons between the two limbs.^29,31,40,41,45,48^ Two articles reported significant lower peak torques for isometric hip flexion in the residual limb compared to the intact limb.^31,48^ For isometric hip extension, three articles reported significantly lower peak torques in the residual limb.^29,31,48^ Isokinetic hip flexion and extension torque were assessed in four articles.^31,40,41,48^ The residual limb demonstrated significantly lower peak isokinetic hip flexion torques compared to the intact limb, across multiple angular velocities.^40,48^ Similarly, the peak isokinetic hip extension torque was significantly lower in the residual limb compared to the intact limb.^40,48^ Two articles reported non significant differences in hip flexion torque,^29,41^ and one reported non significant differences in hip extension torque.^41^ In contrast to the other studies, these articles did not normalize peak torque to body mass. Articles that did not statistically analyze between-limb differences generally showed lower or comparable hip flexion and extension torques in the residual versus the intact limb.^43,44^ In contract, Sawers & Fatone^45^ found significantly higher peak isometric hip flexion and extension torques in the residual limb after normalization to body mass and thigh length and a log-transform.

Five articles reported hip flexion and extension torque for the residual and a control limb.^28,29,35,45,50^ Heitzmann et al.^28^ observed a significant 43% reduction in peak isometric hip flexion torque in the residual limb versus a control limb after normalizing to body mass, along with a non-significant 15% reduction in peak isometric hip extension torque. Two other articles also described reductions in peak isometric hip flexion and extension torque in the residual limb compared to a control limb, although not statistically tested.^29,35^ Sawers & Fatone^45^ found no significant between‑limb differences in isometric peak hip flexion or extension torque when comparing the intact with a control limb. For individuals with a bilateral amputation, Visser et al.^50^ found significantly lower isokinetic peak extension torque compared to controls during concentric contractions at 30°/s. However, no significant differences were observed for hip flexion or eccentric contractions.^50^

### Hip Abduction and Adduction

Nine articles reported hip abduction and adduction torque in the residual and intact limb,^29,32,37,38,40,44,45,47,48^ of which four assessed only hip abduction.^32,37,44,47^ Five articles directly compared both limbs.^29,32,40,45,48^ For isometric hip abduction and adduction, one article reported a significant lower peak torque in the residual limb for hip abduction but not adduction.^29^ Another article assessed only adduction torque and reported a significant deficit,^32^ while a third article found no statistical differences in isometric peak torque between the residual and intact limb for abduction or adduction.^48^ In contrast, Sawers & Fatone^45^ found significant higher peak abduction and adduction torques for the residual limb after normalizing to body mass and thigh length.

Three articles examined isokinetic hip abduction and/or adduction torque.^32,40,48^ Peak isokinetic hip abduction torque was consistently lower in the residual limb across all tested velocities.^40,48^ For hip adduction, only the 30°/s condition showed a significantly lower residual-limb torque.^40,48^ Studies without statistical comparisons generally showed lower residual-limb torques for hip abduction,^32,37,47^ except for Sawers & Fatone,^38^ who reported higher abduction and adduction torques for the residual limb compared to the intact limb after normalizing to body mass and thigh length.^38^

Six articles reported peak isometric hip abduction and/or adduction torque of the residual limb and a control limb.^28,29,32,35,37,45^ Heitzmann et al.^28^ reported significantly reduced peak isometric hip torque in the residual limb compared to the control limb, with a 40% reduction in abduction and a 36% reduction in adduction.^28^ However, two other articles reported non-significant differences in isometric abduction torque between the residual and a control limb,^32,45^ although they included both individuals with a TFA and TTA. Three articles did not statistically analyze the differences but showed a lower residual-limb abduction and adduction torque compared to controls, during isometric testing.^29,35,37^ For isometric hip adduction, Sawers & Fatone^45^ reported a significantly higher peak torque in the residual limb compared to controls, again after normalizing to body mass and thigh length. Isokinetic torque was assessed in only two articles, both examining abduction torque. Visser et al.^50^ found no significant between limb differences when comparing bilateral lower-limb amputees with controls, and Ryser et al.^32^ reported reductions in isokinetic hip abduction torque of the residual limb, without statistical testing.

### Muscle Power and Total Work

One study reported significantly lower isokinetic flexion, extension, and abduction muscle power in the residual limb compared to the intact limb, with reductions of 20%-33%.^48^ No significant differences were found for adduction power or for hip extension and abduction power at higher speeds (180°/s and 90°/s, respectively). Another study investigated lower limb muscle power in individuals with unilateral limb loss, comparing fallers, non fallers, and controls.^42^ Fallers demonstrated significantly lower muscle power in their intact limb compared with a control limb, but no statistical comparisons were conducted between the residual limb and the intact or control limb.^42^ Total work, assessed in one study and for flexion and extension only, did not differ significantly between limbs.^41^

## DISCUSSION

This systematic review aimed to provide a comprehensive and critically appraised overview of the existing evidence on muscle morphology and muscle function in the residual limb following TFA. Overall, reductions in muscle cross-sectional area and muscle volume were commonly observed,^33,34,36,39,46,49^ while fat infiltration was less consistently investigated.^30,36,39,46^ Functionally, hip muscle torque, particularly in flexion, extension and abduction, was generally lower in the residual limb compared with the intact limb or control participants,^28,31,32,40,48^ whereas evidence for adduction and isometric torque differences was limited and inconsistent.^29,40,45,48^ Together, these findings indicate substantial but heterogeneous alterations in muscle morphology and function following TFA. As none of the included studies assessed both outcomes concurrently, no conclusions can be drawn regarding their interrelationship, highlighting an important gap in the literature.

The alterations found in muscle morphology following TFA can be attributed to various factors, including changes in mechanical loading and biomechanic conditions.^52^ The reported reduced thigh MCSA, decreased muscle volume in most muscles and increased fatty infiltration in the residual gluteus medius and psoas muscles may reflect reduced use and underloading of the residual limb muscles relative to the intact limb,^52^ a mechanism that can cause substantial reductions in MCSA and muscle volume.^20^ Another plausible explanation is the transition of once-biarticular muscles into monoarticular muscles following amputation, which may alter their functional demands and lead to greater atrophy.^49^ Jaegers et al.^17^ reported that the once-biarticular muscles in the residual limb exhibited greater atrophy (40-60%) compared with the intact muscles in the same limb (0-30%). Inadequate muscle fixation during surgery may also contribute to reductions in muscle volume, as insufficient fixation can lead to muscle retraction and subsequent atrophy,^17^ although conclusions are limited by sparse reporting of surgical techniques across the included studies.

In contrast to general pattern of muscle atrophy, some muscles exhibited increased muscle volume. For example, Yun et al.^33^ reported no significant differences in the adductor muscles and Henson et al.^49^ found significantly increased muscle volumes in the adductor brevis, gluteus medius, and iliopsoas in individuals with a bilateral TFA. These findings align with evidence of increased recruitment of muscles with intact insertions in the residual limb, suggesting compensatory adaptation to functional demands.^52^ Such compensatory mechanisms may lead to selective hypertrophy instead of atrophy.^49^

Besides the morphological changes, functional deficits in hip musculature were also frequently reported. Decreased peak muscle torque of the hip flexors, extensors, abductors, and adductors of the residual limb compared to the intact or control limb was observed in multiple studies.^28,31,32,40,48^ One possible explanation for the reduction in hip abduction torque is the surgical disruption of the insertion of the iliotibial tract,^17^ which may reduce the contribution of the tensor fascia latae and gluteus maximus to hip abduction, as both muscles transmit part of their abduction force through this structure.^53^ Moreover, the reduced muscle volume and increased fat infiltration in the gluteus medius reported by Roda et al.^30^ may also play a role in the diminished hip abduction torque following TFA. Interestingly, reductions in hip adductor torque were reported less consistently than reductions in abductor torque, despite disruption of the adductor magnus insertion following TFA. This may be related to residual limb length as longer residual limbs preserve more adductor muscle and may therefore retain greater strength.^17,18^ However, a modeling study by Ranz et al.^15^ suggests that, as long as the adductor tendon could be reattached, the tension applied during fixation has a greater influence on muscle capacity following TFA than residual limb length. Altogether, this suggests that alterations in muscle function result from a combination of factors rather than a single cause.

As muscle morphology and function are interdependent, both must be examined to fully understand post amputation changes. The reductions in muscle strength after TFA plausibly relate to the reductions in MCSA and muscle volume, given the well-established relationship between muscle size and force-generated capacity in healthy populations.^23^ The loss of muscle mass inherently diminishes the number of contractile units available, resulting in reduced muscle strength and endurance.^54^ Additionally, fatty infiltration exacerbates this issue since the presence of fatty infiltration within muscles has been directly associated with declines in muscle function.^55,56^ However, in atrophied and hypertrophied muscles, the relationship between muscle volume and maximum force changes,^57,58^ due to alterations in muscle characteristics like pennation angle and fiber length.^59^ Furthermore, as previously stated, the method of distal muscle fixation (myodesis vs. myoplasty), the tension (i.e. sarcomere length at neutral angle), and whether muscles are being reattached or not could influence muscle functioning in individuals following TFA.^14–16^ Because none of the included studies assessed both muscle morphology and function simultaneously, and due to substantial study heterogeneity, no quantitative relationship between these outcomes could be established. This highlights an important knowledge gap that should be addressed in future research.

### Clinical Implications

Understanding the muscle alterations and underlying mechanisms is important to improve rehabilitation and surgical outcomes. Previous research has shown that muscle strength deficits following TFA are associated with difficulties in residual limb control, walking performance, and functional mobility.^28,60^ However, it remains unclear how residual muscle adaptations contribute to these strength deficits and functional challenges. Additionally, the extent to which the surgical technique affects the functional capacity of remaining muscles, and thereby the strength they can produce, remains unknown. This limits insight into which muscles can be effectively targeted by proper surgical techniques and in rehabilitation training. The observed variation in muscle morphology and strength changes across muscle groups illustrates the complexity of post-TFA muscle adaptations and emphasizes the need to assess both muscle morphology and function while also considering the surgical technique used. Clarifying how surgical procedures influence morphological and functional muscle changes will provide important insights to guide surgical decision making and help clinicians tailor rehabilitation to optimize functional outcomes.

### Limitations and Recommendations for Future Research

This review offers a comprehensive overview by combining findings on muscle morphology and function following TFA. Nonetheless, some limitations should be considered. First, the quality and number of articles included may have influenced the results and conclusions. Overall, the quality assessment indicated that the majority of articles were of moderate to good quality. However, five articles exhibited lower methodological quality.^29,34,37,43,49^ Despite these shortcomings, it is important to acknowledge the value of these articles given the limited literature available on this topic. In addition, methodological limitations related to the literature search should be acknowledged. Language and database restrictions may have resulted in missed publications, though the impact is likely limited given the scarcity of literature.

It is important to note that this review may have overlooked the potential influence of demographic, amputation-related, and activity-related factors on muscle morphology and function. For amputation-related factors, the potential impact of the residual limb length and variations in surgical procedures has already been described above. Only two studies reported on the presence or absence of hip contractures,^28,32^ limiting insight into their prevalence and potential influence on muscle outcomes. The time since amputation may also play a role, as muscle atrophy, reorganization and responsiveness to rehabilitation can evolve over time. In this review, time since amputation ranged from 1 month to 48 years, which may have influenced the variability in study outcomes. Beyond amputation-related factors, demographic variables such as age and sex, as well as lifestyle and activity levels before and after amputation may influence the degree of muscle adaptation. Future research should more systematically identify and control such factors to improve the rigor of study designs and deepen our understanding of how amputation affects muscle morphology and function.

Lasty, the heterogeneity of the study populations and measurements limits the generalizability of the findings across different subgroups of individuals with a TFA. Although this review offers valuable insights into the broader TFA population, the inclusion of articles with both TFA and TTA participants, as well as two articles that included only individuals with bilateral amputations, may have influenced the outcomes and reduced the specificity of the conclusions for those with a TFA. However, restricting inclusion to articles with separate TFA reporting would have resulted in the exclusion of 10 more articles. This trade-off between specificity and completeness should be considered when interpreting the findings of this review.

Additional heterogeneity was present in the measurement and reporting of muscle morphology and muscle torque. Articles assessing muscle morphology used different methods to calculate the percentage of atrophy. While some articles compared the residual limb measures with the whole intact or control limb,^34,49^ others restricted their comparison to the portion of the intact limb that matched the level of amputation.^17^ One study additionally normalized muscle volume to body mass and height.^30^ Furthermore, articles evaluating muscle torque differed in their use of handheld versus instrumented dynamometry, as well as in their approaches to normalizing torque values. Sawers & Fatone^45^ normalized hip torque to body mass and thigh length, which resulted in substantially higher torques in the residual limb compared to the intact limb in all movement directions. This contrasts with studies that normalized hip torque to body mass alone, which generally reported lower torques in the residual limb than the intact or a control limb. Such variations make it difficult to compare articles and underscores the need for standardized methods for reporting muscle atrophy and torque. This heterogeneity also precluded quantitative synthesis, including meta-analysis. Altogether, future research should take into account potential confounders and should investigate muscle morphology and function together to enhance our understanding of how a TFA affects muscle morphology and function.

## CONCLUSION

The currently available literature demonstrates reduced MSCA, muscle volume, and muscle strength in the residual limb following TFA. However, the magnitude of these reductions varies in muscle groups and across articles, reflecting the complexity of muscular adaptations after amputation. Future studies should account for potential influencing factors and consider the surgical technique used to deepen our understanding of how a TFA affects muscle morphology and function. Greater standardization of outcome measures is essential to enable meaningful comparisons across articles. Importantly, future studies should also combine assessments of muscle morphology and function to better clarify how structural adaptations relate to functional changes following TFA.

## Appendix: Search Strategies

### PubMed:

((“Muscle Strength”[Mesh] OR “Muscular Atrophy”[Mesh] OR “Muscle*”[tiab] OR “strength” [tiab] OR “morphology” [tiab]) AND ((“Amputation, Surgical”[Mesh] AND “Lower Extremity”[Mesh]) OR “lower limb amput*”[tiab] OR “trans-femoral amput*”[tiab] OR “transfemoral amput*”[tiab] OR “amputated limb”[tiab] OR trans femoral amput*[tiab] OR “above knee amput*”[tiab])) NOT (“transtib*”[ti] OR “Systematic Review” [Publication Type] OR “Meta-Analysis” [Publication Type])

### Embase:

('muscle*':ti,ab OR 'strength':ti,ab OR 'morphology':ti,ab) AND ('lower limb amput*':ti,ab OR 'trans-femoral amput*':ti,ab OR 'transfemoral amput*':ti,ab OR 'amputated limb':ti,ab OR 'trans femoral amput*':ti,ab OR 'above knee amput*':ti,ab) NOT ('systematic review':pt OR 'meta-analysis':pt)

### CINAHL:

((Muscle* OR strength OR morphology) AND (lower limb amput* OR trans-femoral amput* OR transfemoral amput* OR amputated limb OR “trans femoral amput*” OR “above knee amput*”)) NOT (Publication Type: “Systematic Review” OR Publication Type: “Meta-Analysis”
